# Effects of prescribed fire and social insects on saproxylic beetles in a subtropical forest

**DOI:** 10.1038/s41598-020-66752-w

**Published:** 2020-06-15

**Authors:** Michael D. Ulyshen, Andrea Lucky, Timothy T. Work

**Affiliations:** 1 USDA Forest Service, Southern Research Station, Athens, GA USA; 2 Entomology and Nematology Department, University of Florida, Gainesville, FL USA; 3 Département des Sciences Biologiques, Université du Québec à Montréal, Montreal, Canada

**Keywords:** Ecology, Ecology

## Abstract

We tested the immediate and delayed effects of a low-intensity prescribed fire on beetles, ants and termites inhabiting log sections cut from moderately decomposed pine trees in the southeastern United States. We also explored co-occurrence patterns among these insects. Half the logs were placed at a site scheduled for a prescribed fire while the rest were assigned to a neighboring site not scheduled to be burned. We then collected insects emerging from sets of logs collected immediately after the fire as well as after 2, 6, 26 and 52 weeks. The fire had little effect on the number of beetles and ants collected although beetle richness was significantly higher in burned logs two weeks after the fire. Both beetle and ant communities differed between treatments, however, with some species preferring either burned or unburned logs. We found no evidence that subterranean termites (*Reticulitermes*) were influenced by the fire. Based on co-occurrence analysis, positive associations among insect species were over two times more common than negative associations. This difference was significant overall as well for ant × beetle and beetle × beetle associations. Relatively few significant positive or negative associations were detected between termites and the other insect taxa, however.

## Introduction

Dead wood is one of the most important resources for biodiversity in forests worldwide, supporting diverse and interacting assemblages of invertebrates, fungi and prokaryotes^1,2^. In addition to many species that opportunistically benefit from dead wood, about one third of all forest insect species are saproxylic, meaning they are strictly dependent on this resource at some stage in their life cycle, and many of them have become threatened in intensively managed landscapes^3,4^. Hundreds of studies aimed at conserving this fauna have been conducted over the past several decades, with particular attention paid to the effects of forest management activities and natural disturbances. Many facets of this vast topic remain poorly studied, however, and there is a particular need for research in subtropical and tropical forests^5^. The main objective of this study was to test the immediate and delayed effects of prescribed fire on wood-dwelling insects in the southeastern United States. A second objective was to explore patterns of co-occurrence among wood-dwelling insects, with a particular focus on how social insects affect the diversity and composition of saproxylic insect communities.

Fire is an important natural disturbance in many forests and is commonly prescribed by forest managers as a way to reduce the risk of wildfire, create more open forest conditions and to stimulate the growth of understory vegetation. Numerous studies have investigated the effects of fire on saproxylic insects and most of these have shown general benefits to biodiversity^6^. However, interpretation of these patterns is complicated by the fact that most studies have involved using traps to sample flying insects and the sunnier and warmer conditions following fires may stimulate insect activity in burned forests. By contrast, very few studies have attempted to sample insects directly from dead wood following a burn. Wikars^7^ sampled insects from burned and unburned spruce and birch logs in Sweden and found bark beetles to be negatively affected by fire whereas fungus feeders responded positively due to the beneficial effects of the fire on ascomycete fungi. In the southeastern United States, Ulyshen *et al*.^8^ reported less than half as many beetle individuals from burned vs. unburned logs but the number of species was unaffected. Similar results were shown in Canada by Boucher *et al*.^9^ who collected nearly five times more beetles from unburned wood than from wood burned in a wildfire but saw no difference in species richness between burn treatments. Similar to Wikars^7^, this latter study found some species to be strongly associated with burned logs while others were more strongly associated with unburned wood. Many insects have become fire-adapted in response to frequent burns. While many of these benefit from fire but do not depend on it, others are truly pyrophilous, meaning they have evolved traits in response to fires and suffer population declines in the absence of fire^6^. Due to this close association, pyrophilous species are sensitive to fire frequency and have been shown to be rare following long periods of fire suppression. For example, Johansson *et al*.^10^ captured very few fire-dependent beetles following a large wildfire in northern Sweden where there had been over a century of fire suppression. Relatively little is known about the diversity and conservation status of pyrophilous insects outside of Europe, however.

While interspecific interactions among saproxylic insects have received a lot of attention, past work has focused largely on the antagonistic interactions among bark beetles^11^. In this study, we were particularly interested in better understanding how social insects (i.e., termites and ants) affect the composition of saproxylic insect communities as this is a recognized knowledge gap in the literature^5^. Social insects are often extremely abundant in dead wood^12,13^, especially in tropical/subtropical forests^14^, but very little is known about how they interact with other members of the saproxylic community. Ants are largely predatory and may, in turn, negatively affect populations of many wood-dwelling insect species, including termites^15,16^. By dominating food resources and space, termites may exclude other insects from dead wood, with potentially negative effects on saproxylic insect diversity.

In this paper, we present the results pertaining to the two objectives separately. For the first objective (i.e., testing the effects of fire), we predicted that fire would have immediate negative impacts on the abundance of beetles, ants and termites and that community composition of burned logs would change over time relative to unburned logs. For the second objective (i.e., investigating co-occurrence patterns), we hypothesized that termites and ants would both have mostly negative effects on beetles and that ants would have mostly negative effects on termites.

## Methods

### Study Area and fauna

The study took place in a mature pine-dominated forest on the Noxubee National Wildlife Refuge in Noxubee County, Mississippi, United States. The climate of the region is classified as humid subtropical with annual precipitation and temperature averaging 141 cm and 16.9 °C, respectively (usclimatedata.com, accessed June 2015). Most of the upland portions of the refuge are regularly burned to maintain loblolly pine (*Pinus taeda* L.) dominance in the canopy and an open understory. Loblolly pine is native to the southeastern United States and is the most widely planted tree species throughout the region^17^. The dead wood produced by this species is known to support hundreds of saproxylic beetle species and other insects^18^. Subterranean termites (Rhinotermitidae) belonging to the genus *Reticulitermes* are among the most abundant insects occurring in wood throughout the region and consume approximately one fifth of wood volume^19^. In addition, there are at least 95 species of ants known from the Noxubee Wildlife Refuge, many of which nest or forage within dead wood^20^.

### Study Design

Twelve previously-fallen loblolly pine (*Pinus taeda*) trees were selected from mature pine-dominated forests. All trees were in contact with the ground and belonged to decay classes 2 or 3 as defined by Woodall and Williams (2005). On 25 March 2011, sixteen log sections (hereafter “logs”) 50 cm in length and 29.7 ± 0.3 cm in diameter were removed from each tree. These logs were numbered sequentially from 1 to 16. Even-numbered logs were placed in a pine-dominated forest scheduled for a prescribed burn whereas odd-numbered logs were placed in an immediately adjacent pine-dominated forest not scheduled for burning, i.e., eight logs from each tree were placed in both the burned and unburned sites. The logs from a given tree were placed in a line and were separated by 5 m. The different rows were also separated by 5 m. To determine the immediate effects of the fire on wood-dwelling insects, one log from each tree and from each site was collected immediately (within one hour) after the fire on 7 April 2011. Additional collections were made after 2, 6, 26 and 52 weeks to explore longer-term effects of fire on wood-dwelling insect communities. For each collection period, one section from each tree was randomly selected from the burned site. One of the logs that had been immediately adjacent to this section in the original tree was then selected from the unburned site. This paired sampling approach was designed to reduce between-log variability in order to better isolate the effect of fire on insect communities.

Although the fire took place in a stand that had not been burned since 2003 and the litter layer was therefore quite thick, a heavy rainstorm several days before the burn resulted in a lower-intensity fire than we had anticipated. Logs that had not been completely encircled in flames (e.g., those in depressions or other relatively low-lying areas) were eliminated from the study. After accounting for this, an effort was made to maximize replication for the early collection periods. All twelve logs were sampled at 0 and 2 weeks, eleven were sampled at 6 weeks and ten were sampled at both 26 and 52 weeks. In total, 110 logs were collected over the course of the study, with different logs collected at each sampling period. The collected logs were returned to the lab and placed in individual emergence bags^21^ to collect any insects that emerged over a period of six months. The collection jars were filled with propylene glycol and captured insects were transferred to ethanol approximately once a month. All beetles were identified to the lowest taxonomic level possible given available time and expertise and abundance data were collected for each log. We recorded the presence or absence of subterranean termites (*Reticulitermes* spp.) from each log. Ants were identified to species level and the presence or absence of each was recorded. The genus *Aphaenogaster* in the southeastern United States includes several morphologically problematic species in the *A. fulva-rudis-texana* complex for which species boundaries are unclear. The use of the names *A. carolinensis* and *A. fulva* are applied based on keys in Umphrey^22^ and DeMarco and Cognato^23^. Similarly, the *Solenopsis molesta-*group thief ants include a number of morphologically indistinguishable species. For simplicity we refer only to *S. molesta* although the ants sampled from this group likely comprise several species in the complex.

### Statistical analysis

To compare beetle (abundance, richness, diversity and NMDS values for axes 1–3 (see below)) and ant (richness and NMDS values for axes 1–3 (see below)) metrics between burned and unburned logs, we ran mixed models in SAS 9.4^24^ consisting of treatment, time and the treatment × time interaction as fixed effects and tree as a random effect. We initially also included terms for termites (presence/absence) and log diameter but these were both nonsignificant in all models and therefore dropped from the final analyses. To test whether termite (*Reticulitermes*) incidence (i.e., presence/absence) differed between burned and unburned logs, Fisher’s exact test was conducted on each time period separately as well as on all time periods combined. Nonmetric multidimensional scaling, using the “slow-and-steady” autopilot mode in PC-ORD ver. 6^25^, was performed on the beetle and ant datasets to investigate differences in community composition between burn treatments and among sampling periods. In both cases, all species appearing in fewer than three samples were excluded from the analysis. The Jaccard distance measure was used for ant presence/absence data and the Bray-Curtis distance measure was used on beetle abundance data after relativizing by species maxima (i.e., to better equalize the influence of common and rare species). Indicator species analyses were carried out on the same datasets to identify species significantly associated with burned or unburned substrates. Indicator values (IV) were calculated based on the following equation: IV_ij_ = A_ij_ × B_ij_ × 100 where A_ij_ is the mean abundance of species i in logs of group j compared to all groups and B_ij_ is the relative frequency of occurrence of species i in logs of group j^26^. Indicator values range from 0 (no indication) to 100 (perfect indication) and reflect how abundant a particular category is in one group compared to other groups as well as the constancy of that species within a group. Significance was determined by a Monte Carlo randomization test with 4999 permutations.

To determine if beetle and ant communities were significantly correlated, we used PC-ORD^25^ to conduct both Mantel tests and partial Mantel tests. Following Smouse *et al*.^27^, partial Mantel tests include a third matrix to control for environmental variables (treatment, time, diameter, tree and termite incidence). The same modified matrices used in the NMDS analyses (see above) were tested first and these results were compared with those based on unmodified matrices. The Jaccard distance measure was used for ant presence/absence data and the Bray-Curtis distance measure was used on beetle abundance data. To examine the relationship between the richness of beetles and ants, we ran a mixed model consisting of beetle richness as the response, ant richness and time as fixed effects and tree (1–12) as a random effect. Finally, to look at pairwise associations between insect species sampled in this study, we performed co-occurrence analysis using the package cooccur^28^ in R 3.6.1^29^. Beginning with a complete matrix of all 259 species and 110 logs, only species pairs with an expected co-occurrence ≥ 1 were retained for analysis with expected co-occurrence being the product of the two species’ probability of occurrence times the number of sampling units^28^. For each of these retained pairs, the function returns probabilities that the two species could co-occur less than or greater than what is observed in the data. Following Griffith *et al*.^28^, these are reported here as P-values, indicating significance levels for negative and positive associations, respectively. To visualize these patterns, observed-expected plots^28^ were prepared for five pair combinations: termite × ant, ant × ant, termite × beetle, ant × beetle and beetle × beetle. When one of these pair combinations contained more than ten significant associations, a Chi Square test was used to determine if the relative number of negative and positive associations differed significantly from a 1:1 ratio. This test was also performed on all significant associations combined.

## Results

### Insect emergence

In total, 12,866 beetles belonging to 220 species (or morphospecies) emerged from the 110 logs collected in this study. Burned logs yielded 6,501 individuals and 187 species while 6,365 individuals and 145 species emerged from unburned logs. The number of beetle species collected varied greatly among logs, ranging from 3–35. A total of 38 ant species emerged from the logs, with 34 and 35 species recovered from burned and unburned logs, respectively (Table 1). As with beetles, ant richness varied greatly among logs, ranging from 1–8 species. Termites were active in 73 of the 110 logs (66%), about equally divided between burned (36) and unburned (37) logs.

**Table 1:** List of ant species collected in this study and the percent log occupancy for each by burn treatment and in total.

Species	Percent logs occupied		
	Burned (n = 55)	Unburned (n = 55)	Total (n = 110)
*Aphaenogaster carolinensis* Wheeler	10.9	23.6	17.3
*Aphaenogaster fulva* Roger	32.7	54.5	43.6
*Aphaenogaster lamellidans* Mayr	14.5	9.1	11.8
*Camponotus castaneus* (Latreille)	5.5	5.5	5.5
*Camponotus chromaoides* Bolton	12.7	14.5	13.6
*Camponotus decipiens* Emery	0	1.8	0.9
*Camponotus subbarbatus* Emery	25.5	45.5	35.5
*Crematogaster ashmeadi* Mayr	3.6	3.6	3.6
*Crematogaster cerasi* (Fitch)	5.5	1.8	3.6
*Crematogaster lineolata* (Say)	3.6	0	1.8
*Crematogaster pilosa* Emery	1.8	0	0.9
*Cryptopone gilva* (Roger)	1.8	3.6	2.7
*Dorymyrmex flavus* McCook	1.8	1.8	1.8
*Hypoponera opaciceps* (Mayr)*	1.8	1.8	1.8
*Hypoponera opacior* (Forel)	10.9	9.1	10
*Lasius alienus* (Foerster)	10.9	18.2	14.5
*Lasius umbratus* (Nylander)	0	1.8	0.9
*Monomorium minimum* (Buckley)	63.6	45.5	54.5
*Myrmecina americana* Emery	5.5	7.3	6.4
*Nylanderia faisonensis* (Forel)	40	27.3	33.6
*Nylanderia querna* Kallal and LaPolla	14.5	5.5	10
*Pheidole dentata* Mayr*	34.5	9.1	21.8
*Pheidole navigans* Forel*	0	7.3	3.6
*Ponera exotica* Smith, M.R.	1.8	3.6	2.7
*Ponera pennsylvanica* Buckley	9.1	16.4	12.7
*Proceratium croceum* (Roger)	9.1	3.6	6.4
*Proceratium pergandei* (Emery)	1.8	0	0.9
*Proceratium silaceum* Roger	7.3	9.1	8.2
*Solenopsis geminata* (Fabricius)*	9.1	3.6	6.4
*Solenopsis molesta* (Say)	50.9	52.7	51.8
*Stigmatomma pallipes* (Haldeman)	0	1.8	0.9
*Strumigenys clypeata* Roger	7.3	3.6	5.5
*Strumigenys louisianae* Roger	5.5	10.9	8.2
*Strumigenys ornata* Mayr	3.6	1.8	2.7
*Strumigenys rostrata* Emery	16.4	40	28.2
*Temnothorax curvispinosus* (Mayr)	38.2	12.7	25.5
*Temnothorax pergandei* (Emery)	1.8	1.8	1.8
*Temnothorax schaumii* (Roger)	1.8	1.8	1.8

Asterisks indicate non-native species.

### Fire effects

**Figure 1:**
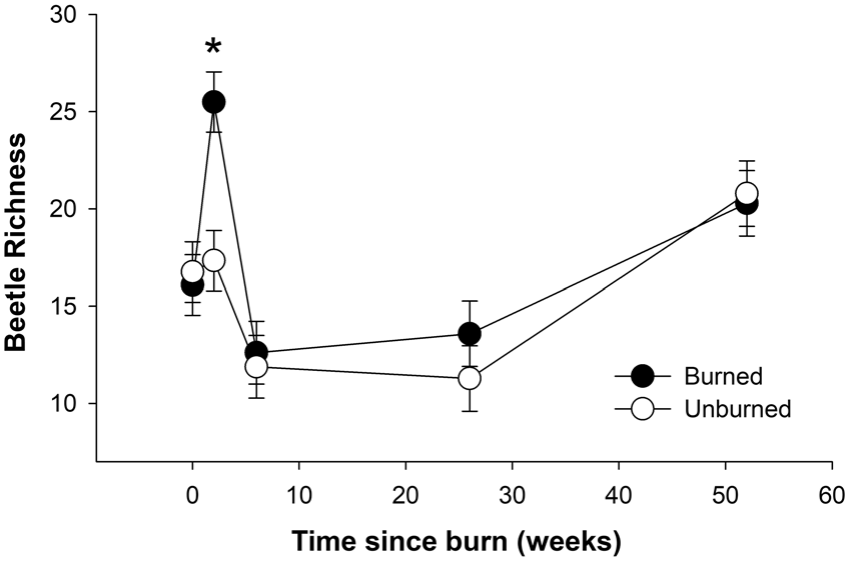
Least square means ± SE beetle richness over time. Asterisk indicates a significant difference between treatments at 2 weeks but not at any other time period.

Total beetle abundance and beetle diversity both changed over time but were unaffected by treatment and there were no significant interactions between these terms for either metric (Table 2). Beetle richness varied with time and between treatments and there was a significant treatment × time interaction. The interaction was due to a significantly greater number of beetle species in burned vs. unburned logs at two weeks but no differences between treatments for any other time period (Fig. 1). There were significant differences between treatments for beetle NMDS axes 1 and 3 and among time periods for axes 2 and 3. There were no significant interactions between treatment and time, however (Table 2). Based on indicator species analysis, eight beetle species were significantly associated with burned logs while three others were significant indicators of unburned logs (Table 3). When indicator species analysis was performed on beetle data from the 2 week collection period, two carabid taxa (*Paratachys* (IV = 66.7, P = 0.0016) and *Polyderis laevis* (IV = 75.3, P = 0.002)) and three staphylinid taxa (*Microscydmus* (IV = 58.3, P = 0.0058), *Lobrathium* (IV = 50, P = 0.013) and *Palaminus* (IV = 50, P = 0.014)) were significantly associated with burned logs. Ant richness did not vary between treatments or with time and there was no treatment × time interaction. Ant community composition did differ between treatments based on all three NMDS axes and there were no treatment × time interactions for these metrics (Table 2). Based on indicator species analysis, two ant species were significantly associated with burned logs while three others were significant indicators of unburned logs (Table 3). Based on Fisher’s exact tests, there were no differences in termite (*Reticulitermes* spp.) incidence between burned and unburned logs for any of the time periods or when all time periods were combined.

**Table 2:** Results from mixed models with asterisks denoting significance (*P < 0.05, **P < 0.001, ***P < 0.0001).

	Beetle abundance	Beetle diversity	Beetle richness	Beetle axis 1	Beetle axis 2	Beetle axis 3	Ant richness	Ant axis 1	Ant axis 2	Ant axis 3
treatment	F_1,89.5_ = 0.99	F_1,87.3_ = 2.06	F_1,89.4_ = 5.03*	F_1,89.5_ = 6.39*	F_1,88.8_ = 0.84	F_1,89.2_ = 6.72*	F_1,100_ = 0.0	F_1,89.4_ = 8.22*	F_1,100_ = 7.39*	F_1,100_ = 18.89***
time	F_4,90.6_ = 11.56***	F_4,89.5_ = 4.35*	F_4,90.5_ = 18.48***	F_4,90.7_ = 1.81,	F_4,89.2_ = 42.08***	F_4,89.8_ = 11.47***	F_4,100_ = 1.92	F_4,91.3_ = 3.46*	F_4,100_ = 1.6	F_4,100_ = 12.73***
treatment*time	F_4,89.5_ = 1.32	F_4,87.3_ = 1.06	F_4,89.4_ = 3.56*	F_4,89.5_ = 1.91	F_4,88.8_ = 0.35	F_4,89.2_ = 1.82	F_4,100_ = 0.46	F_4,89.4_ = 2.31	F_4,100_ = 1.16	F_4,100_ = 0.39

Termites (present/absent) and log diameter were non-significant and thus removed from all models.

**Table 3:** Ants and beetles found to be significantly associated with burned or unburned logs based on indicator species analysis.

Family	Species	Burned	Unburned
Formicidae	*Pheidole dentata* Mayr	IV = 27.3, P = 0.002	
	*Temnothorax curvispinosus* Mayr	IV = 28.6, P = 0.004	
	*Strumigenys rostrata* Emery		IV = 28.4, P = 0.013
	*Aphaenogaster fulva* Roger		IV = 34.1, P = 0.034
	*Camponotus subbarbatus* Emery		IV = 29.1, P = 0.039
Carabidae	*Clivina pallida Say*		IV = 16.5, P = 0.031
	*Paratachys* sp.	IV = 18.2, P = 0.002	
	*Polyderis* sp.	IV = 24.5, P = 0.006	
Ciidae	spp.	IV = 33.3, P = 0.046	
Staphylinidae	*Microscydmus* sp.	IV = 12.7, P = 0.015	
	Aleocharinae sp.		IV = 13.7, P = 0.021
	*Lobrathium* sp.	IV = 12.7, P = 0.011	
	*Palaminus* sp.	IV = 10.9, P = 0.024	
	*Melba thoracica* (Brendel)	IV = 17.8, P = 0.022	
Tenebrionidae	*Platydema nigratum* (Mots.)		IV = 10.9, P = 0.029
	*Uloma punctulata* LeConte	IV = 35.6, P = 0.04	

### Insect interactions

Mantel tests indicate beetle and ant communities were not significantly correlated when rare species were dropped from the matrices and species abundances were relativized by maxima (r = 0.03, P = 0.28) but were significantly correlated when the unmodified matrices were compared (r = 0.08, P = 0.01). The respective results for the partial Mantel tests were the same: non-significance when reduced matrices were used (r = 0.01, P = 0.27) but a significant correlation when the unmodified matrices were compared (r = 0.05, P = 0.04). Our model testing the relationship between beetle richness and ant richness found a significant positive relationship (F_1,102_ = 16.9, P < 0.0001).

**Figure 2:**
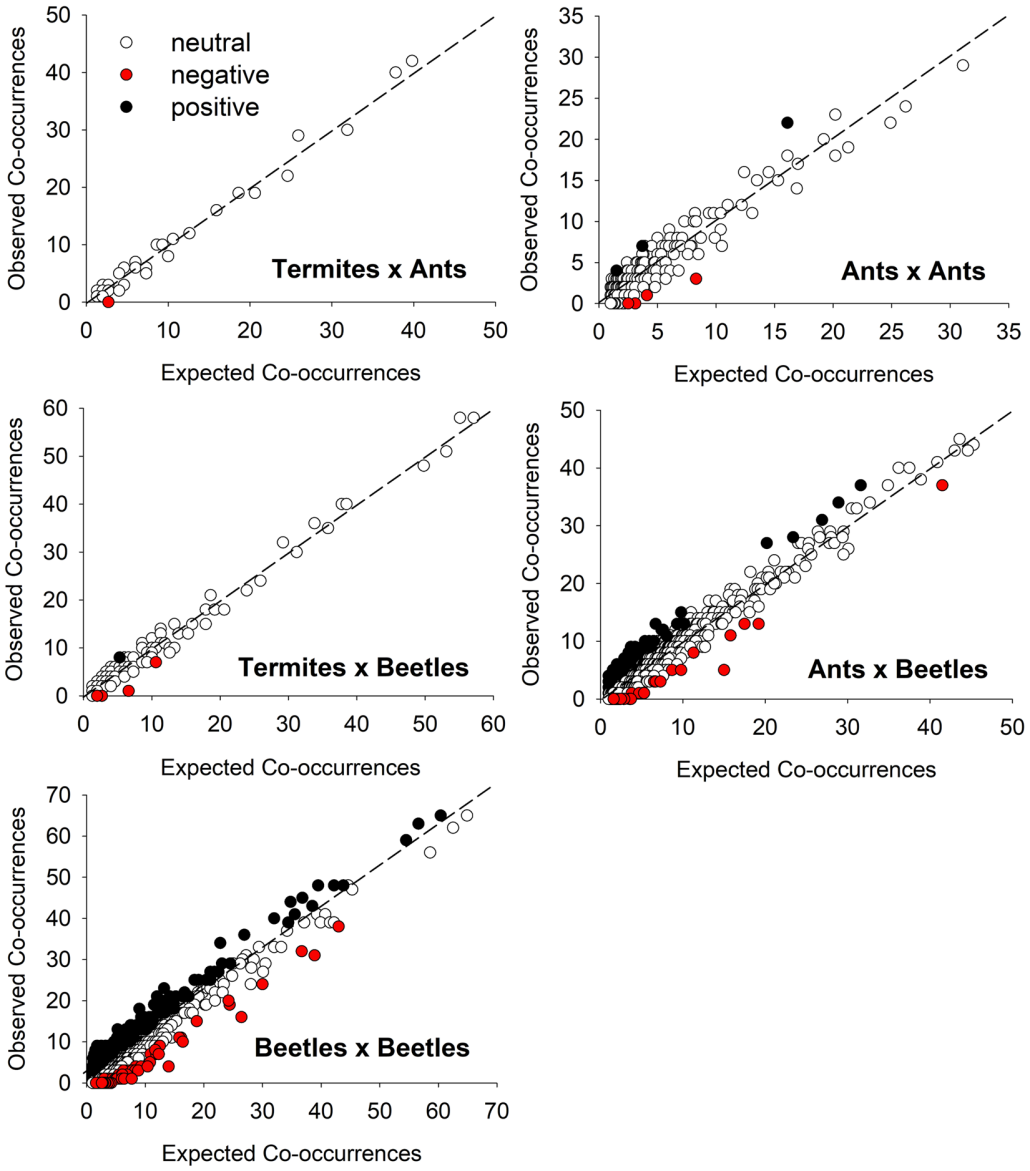
Observed-expected plots showing the relative numbers of positive and negative associations between species pairs based on co-occurrence analysis.

There were 33,411 possible species pair combinations among the 259 species included in the co-occurrence analysis. Of these, 28,551 (85.45%) pairs were eliminated because expected co-occurrence was <1, leaving 4,860 combinations for the analysis. Of these analyzed combinations, there were 300 (6.2%) positive and 116 (2.4%) negative associations while the remaining 4,444 (91.4%) were classified as random (hereafter “neutral”) (Table 4). Overall, there were significantly more positive than negative associations (χ^2^(1, *N* = 416) = 81.38, P < 0.0001) and this was also true for ant × beetle associations (χ^2^(1, *N* = 100) = 17.64, P < 0.0001) as well as beetle × beetle associations χ^2^(1, *N* = 301) = 73.76, P < 0.0001 (Fig. 2). The other pair groupings had too few significant associations to test the relative number of negative and positive associations. When looking at associations with individual species, positive co-occurrence patterns predominated, with only 11.7% of species showing more negative than positive associations (Supplementary Table S2). One ant (*Pheidole navigans* Forel) and five beetle species (*Chalcophora virginiensis* (Drury), *Conalia helva* (LeConte), *Sepedophilus macer* (Casey), *Vanonus*, *Xyleborus bispinatus* Eichhoff) were negatively associated with termites whereas only a single species of beetle, an unidentified staphylinid, was positively associated (Supplementary Table S1).

**Table 4:** Summary of results from co-occurrence analysis separated by various pair categories: termite × ant, ant × ant, termite × beetle, ant × beetle and beetle × beetle.

Combinations	Co-occurrence pattern			
	Neutral	Negative	Positive	Total
termite × ant	32	1	0	33
ant × ant	217	5	3	225
termite × beetle	140	5	1	146
ant × beetle	1566	29	71	1666
beetle × beetle	2489	76	225	2790
Total	4444	116	300	4860

## Discussion

The main objectives of this study were to explore the immediate and delayed effects of a prescribed fire on wood dwelling insects and to investigate patterns of co-occurrence among beetles, ants and termites (*Reticulitermes* spp.). As discussed below, our findings indicate that insects in dead wood are highly resilient but still somewhat affected by low-intensity ground fires in the southeastern United States. In addition, our results answer some long-standing questions about the degree to which ants, termites and beetles coexist within dead wood.

### Fire effects

While fire had no overall effect on beetle abundance or diversity, there was a significant interaction between treatment and time for beetle richness. Richness was significantly higher in burned logs two weeks after the burn, suggesting there was a rapid colonization of logs following the fire. While the loss of other forms of shelter from the forest floor may have contributed to this movement into the logs, the results from indicator species analysis suggest that the increase in richness was largely driven by the movement of opportunistic predators into the logs. Whatever the cause, this increase in richness was short lived, as there were no treatment differences at any subsequent point in time. We also found beetle community composition to differ significantly between burn treatments based on two of the three NMDS axes tested. Based on indicator species analysis, 8 species showed a significant association with burned logs while three others were associated with unburned logs. These findings support the notion that some saproxylic insect species prefer burned substrates. While these species may be favored by fire, none of them, to our knowledge, is fire-dependent. Moreover, there was no overlap between apparently fire-favored species in this study and the one previous study to address this question in the southeastern United States. Overall, the beetle community in this study was much less affected by fire than in the previous study by Ulyshen *et al*.^8^ in which a > 50% reduction in total beetle abundance was reported. This discrepancy is most likely due to fire intensity being considerably lower in the current study. Despite finding a reduction in total beetle abundance, the study by Ulyshen *et al*.^8^ found little evidence that beetles are eliminated by prescribed fire. The only species highlighted in that study as possibly eliminated by fire was *Diplocoelis rudis* (LeConte) which was collected from multiple burned logs in this study. While these studies suggest a high degree of resilience to fire among saproxylic beetles, repeated fires over long periods of time may nevertheless have important effects on the composition of saproxylic beetle assemblages.

Based on presence/absence data, we detected no overall effect of fire on termites in this study. These findings are consistent with previous studies that have shown these insects to be highly resilient to fire in the southeastern United States^30^. Such patterns are not too surprising considering subterranean termites spend a lot of time below ground where they would be well insulated from the heat of the fire. The movement of these insects between the soil and above-ground dead wood apparently makes them highly tolerant of a wide variety of environmental stresses. Previous work has found them to persist even in forests that annually experience prolonged flooding, for example^31^. While our results suggest low-intensity fires have little effect on termites, the situation may be different in places experiencing more intense fires. Abensperg-Traun and Milewski^32^, for example, reported lower wood-feeding termite abundance and diversity two years after an extremely intense fire (preceded by 30 years of fire suppression) in western Australia, possibly resulting from the destruction of colonies within burned wood. Moreover, charred wood was less readily utilized by termites in eastern Australia than unburned wood^33^, suggesting fires can reduce food quality for these insects.

While the fire had no overall effects on ant richness in this study, there were significant differences in ant community composition between fire treatments based on all three NMDS axes. According to indicator species analysis, three species were significantly associated with unburned logs while two others were more common in burned logs. Much like the results for beetles, these findings suggest that the effects of fire vary greatly among species. The same conclusion was reached in a recent study of litter-dwelling ants in Florida where ant community composition varied significantly between frequently-burned and long-unburned longleaf pine (*Pinus palustris* Mill.) flatwoods^34^. Ant species density was lower in frequently-burned plots than in long-unburned plots, suggesting this fauna may be sensitive to high burn frequency. In Canada, Boucher *et al*.^35^ compared ant colonization of wood across a 60-yr post-fire chronosequence. Ant abundance increased for the first 30 years and then declined. The most common ant species in that study was absent the first two years following a fire, further suggesting short-term negative effects on some species.

### Insect interactions

To our knowledge, this is the first study to explore relationships among termites, ants and beetles in dead wood. Overall our results indicate that positive patterns of co-occurrence are more common than negative patterns, suggesting that antagonistic interactions among these species are relatively uncommon. Beetle and ant communities were significantly correlated based on Mantel tests on the full data, and there was a positive correlation between beetle richness and ant richness. There were significantly more positive co-occurrences between ants and beetles than negative ones. These results were unexpected considering the predatory habits of many ant species but may reflect specialized diets of many ant species which do not prey on termites and beetles. There are a number of other possible explanations for these patterns. For example, it is possible that ants preferentially colonize logs with the highest diversity of potential insect prey but most species are deep within the wood and thus inaccessible to them. It is also possible that beetles facilitate the colonization of wood by ants. Holes and abandoned galleries created by wood-boring insects, for example, are known to provide access or nesting space for other insects, including ants^16,36,37^. Finally, diversity patterns for different insect groups may generally be positively correlated as some logs simply provide better resources due to patterns of fungal colonization and stages of decay. Similarly, few negative associations were detected between ants and termites despite the fact that ants are among the most important natural enemies of termites^16^. The ability of these two groups to coexist within the same logs can probably be attributed to the fortifications constructed by termites^38^. One ant species, however, was negatively associated with termites. *Pheidole navigans* is a non-native generalist, preying on live or dead arthropods as well as seeds and nectar. Their colonies can grow quite large (up to 600 individuals) and they are known to forage close to their nest site^39^. Non-native ant species often dominate ant communities within their introduced range, often resulting in severe reductions in the abundance of certain species and overall reductions in native ant biodiversity (Holway *et al*.). Finally, five of the six significant associations detected between termites and beetles were negative. Because active termite colonies can occupy substantial amounts of space within dead wood, it is possible that these patterns are driven by reductions in the amount of habitat available to other insects.

## Conclusions

Two main conclusions can be reached from this study. First, the low intensity prescribed fires employed across the southeastern United States appear to have minimal direct effects on wood-dwelling beetles, ants and termites but responses at the species level are variable. Repeated fires over long periods of time have the potential to result in significant changes to saproxylic insect assemblages. Second, interactions among these three groups of insects are primarily neutral or positive in nature, with negative interactions being significantly less common. It should be noted that this study was based on just one fire at a single site in the southeastern United States. The effects of fire on wood-dwelling insects are likely to vary greatly depending on a variety of site and fire-specific parameters including fire intensity, frequency and seasonality. Despite this limitation, this study provides valuable insights into how fire impacts insects inhabiting dead wood as well as patterns of co-occurrence among this fauna. Future studies testing these questions with more intense fires or in less fire-adapted systems would be of great interest.

## Supplementary information

Supplementary Information.
