# Supplementary material for: Effects of prescribed fire and social insects on saproxylic beetles in a subtropical forest
Source: Sci Rep. 2020 Jun 15;10:9630. doi: 10.1038/s41598-020-66752-w (PMC7295812; doi:10.1038/s41598-020-66752-w)
Supplement: Supplementary file 1 — Supplementary Information. [file 41598_2020_66752_MOESM1_ESM.pdf]

Supplementary Information

Effects of prescribed fire and social insects on saproxylic beetles in a subtropical forest

Michael D. Ulyshen, Andrea Lucky, Timothy T. Work

Supplemental Table S1. Results from co-occurrence analysis showing species pairs found to be significantly negatively or positively associated with one another. The pairs are grouped by termites x ants, ants x ants, termites x beetles, ants x beetles and beetles x beetles and are within these groups sorted in order of increasing positive association.

Combination	Species 1	Species 2	Obs. no. logs w/ both species	Exp. no. logs w/ both species	Prob. of co-occurrence	Negative association (P)	Positive association (P)
termites x ants	Reticulitermes	Pheidole navigans	0	2.7	0.024	0.011	
ants x ants	Aphaenogaster carolinensis	Aphaenogaster fulva	3	8.3	0.075	0.006	
	Aphaenogaster fulva	Solenopsis geminata	0	3.1	0.028	0.015	
	Camponotus subbarbatus	Solenopsis geminata	0	2.5	0.023	0.042	
	Aphaenogaster carolinensis	Pheidole dentata	1	4.1	0.038	0.044	
	Lasius alienus	Temnothorax curvispinosus	1	4.1	0.037	0.046	
	Aphaenogaster fulva	Nylanderia faisonensis	22	16.1	0.147		0.015
	Hypoponera opacior	Nylanderia faisonensis	7	3.7	0.034		0.033
	Pheidole dentata	Proceratium croceum	4	1.5	0.014		0.039
termites x beetles	Reticulitermes	Chalcophora virginiensis	1	6.6	0.06	0.000	
	Reticulitermes	Xyleborus bispinatus	0	2.7	0.024	0.011	
	Reticulitermes	Conalia helva	0	2	0.018	0.036	
	Reticulitermes	Sepedophilus macer	0	2	0.018	0.036	
	Reticulitermes	Vanonus	7	10.6	0.097	0.040	
	Reticulitermes	Staphylinidae sp. 193	8	5.3	0.048		0.033
ants x beetles	Solenopsis molesta	Sunius confluentus	5	15	0.137	0.000	
	Myrmecina americana	Rhyncolus	0	3.6	0.033	0.005	
	Camponotus chromaoides	Ciidae	1	5.3	0.048	0.009	
	Camponotus subbarbatus	Paratachys	0	3.5	0.032	0.010	

	Camponotus chromaoides	Oxycopis thoracica	0	3.7	0.033	0.010	
	Nylanderia faisonensis	Rhyncolus	13	19.2	0.174	0.011	
	Strumigenys louisianae	Rhyncolus	1	4.7	0.042	0.012	
	Solenopsis molesta	Xylopinus saperdiodes	5	9.8	0.09	0.013	
	Strumigenys rostrata	Priocera castanea	1	5.1	0.046	0.014	
	Solenopsis molesta	Ganascus ventricosus	3	7.3	0.066	0.015	
	Camponotus castaneus	Thoracophorus costalis	0	2.8	0.025	0.021	
	Pheidole dentata	Mioptachys flavicauda	13	17.5	0.159	0.023	
	Myrmecina americana	Pycnomerus haematodes	0	2.8	0.025	0.024	
	Camponotus subbarbatus	Staphylinidae sp. 199	0	2.8	0.026	0.026	
	Solenopsis molesta	Glipostenoda ambusta	3	6.7	0.061	0.027	
	Aphaenogaster carolinensis	Tmesiphorus carinatus	0	2.9	0.027	0.030	
	Aphaenogaster carolinensis	Anacyptus testaceus	0	2.9	0.027	0.030	
	Monomorium minimum	Eustrophopsis bicolor	1	3.8	0.035	0.033	
	Nylanderia faisonensis	Uloma punctulata	11	15.8	0.144	0.038	
	Camponotus chromaoides	Dryopthorus americanus	8	11.3	0.103	0.039	
	Monomorium minimum	Canifa pallipes	0	2.2	0.02	0.040	
	Monomorium minimum	Uloma imberbis	5	8.7	0.079	0.040	
	Aphaenogaster fulva	Melba thoracica	3	6.5	0.06	0.041	
	Camponotus subbarbatus	Diplocoelus rudis	0	2.5	0.023	0.042	
	Camponotus subbarbatus	Lobrathium	0	2.5	0.023	0.042	
	Camponotus subbarbatus	Microscydmus sp. 1	0	2.5	0.023	0.042	
	Strumigenys rostrata	Euvrilleta	0	2.5	0.023	0.044	
	Solenopsis molesta	Mioptachys flavicauda	37	41.5	0.377	0.044	
	Dorymyrmex flavus	Cossonus corticola	0	1.6	0.014	0.046	
	Monomorium minimum	Cossonus corticola	54	46.9	0.426		0.001
	Nylanderia faisonensis	Palaminus	6	2	0.018		0.001
	Nylanderia faisonensis	Paratachys	8	3.4	0.031		0.002
	Nylanderia faisonensis	Polyderis laevis	13	6.7	0.061		0.002
	Pheidole dentata	Tmesiphorus carinatus	9	3.7	0.034		0.002
	Proceratium croceum	Microscydmus sp. 2	6	2	0.018		0.002
	Strumigenys louisianae	Toxidium compressum	6	2	0.018		0.003
	Proceratium croceum	Toxidium compressum	5	1.5	0.014		0.005
	Proceratium silaceum	Uloma punctulata	8	3.8	0.035		0.005
	Camponotus subbarbatus	Rhyncolus	27	20.2	0.184		0.006

	Strumigenys rostrata	Clivina pallida	9	4.2	0.038		0.006
	Monomorium minimum	Priocera castanea	15	9.8	0.089		0.006
	Strumigenys rostrata	Aglyptinus laevis	9	4.2	0.038		0.006
	Nylanderia faisonensis	Lobrathium	6	2.4	0.021		0.006
	Nylanderia faisonensis	Microscydmus sp. 1	6	2.4	0.021		0.006
	Ponera pennsylvanica	Philothermus glabriculus	12	7.4	0.067		0.007
	Ponera pennsylvanica	Euplectus	8	3.6	0.032		0.007
	Camponotus castaneus	Xylopinus saperdiodes	4	1	0.009		0.008
	Camponotus chromaoides	Hymenorus	7	2.9	0.026		0.008
	Camponotus subbarbatus	Scaphisoma sp. 1	7	3.2	0.029		0.009
	Nylanderia faisonensis	Cossonus corticola	34	28.9	0.263		0.010
	Nylanderia faisonensis	Vanonus	10	5.4	0.049		0.011
	Nylanderia faisonensis	Lycidae sp. 104	4	1.3	0.012		0.011
	Nylanderia faisonensis	Staphylinidae sp. 162	4	1.3	0.012		0.011
	Nylanderia faisonensis	Staphylinidae sp. 178	4	1.3	0.012		0.011
	Pheidole dentata	Staphylinidae sp. 199	5	1.7	0.016		0.012
	Strumigenys clypeata	Eblisia carolina	4	1.1	0.01		0.012
	Nylanderia querna	Buprestis lineata	5	1.7	0.015		0.013
	Aphaenogaster fulva	Elateridae sp. 95	5	2.2	0.02		0.014
	Camponotus subbarbatus	Sphindidae	4	1.4	0.013		0.014
	Ponera pennsylvanica	Pycnomerus sulcicollis	4	1.1	0.01		0.015
	Temnothorax curvispinosus	Staphylinidae sp. 214	4	1.3	0.012		0.015
	Camponotus chromaoides	Sunius confluentus	8	4	0.036		0.016
	Nylanderia faisonensis	Carabidae sp. 90	5	2	0.018		0.016
	Strumigenys rostrata	Dryopthorus americanus	28	23.4	0.213		0.017
	Strumigenys louisianae	Clivina pallida	4	1.2	0.011		0.019
	Ponera pennsylvanica	Polyderis laevis	6	2.5	0.023		0.020
	Camponotus subbarbatus	Staphylinidae sp. 189	7	3.5	0.032		0.022
	Strumigenys rostrata	Pityobius anguinus	4	1.4	0.013		0.022
	Ponera pennsylvanica	Clivina pallida	5	1.9	0.017		0.023
	Nylanderia querna	Mioptachys flavicauda	11	8	0.073		0.025
	Ponera pennsylvanica	Eblisia carolina	6	2.7	0.024		0.026
	Strumigenys louisianae	Leptusa	9	6.1	0.056		0.027
	Strumigenys rostrata	Chalcophora virginiensis	6	2.8	0.026		0.029
	Strumigenys rostrata	Ampedus luteolus	12	7.6	0.069		0.030

	Strumigenys rostrata	Eblisia carolina	10	5.9	0.054		0.030
	Monomorium minimum	Philothermus glabriculus	37	31.6	0.288		0.031
	Ponera pennsylvanica	Uloma imberbis	5	2	0.019		0.031
	Aphaenogaster lamellidans	Cossonus corticola	13	10.2	0.092		0.033
	Camponotus subbarbatus	Piesmus submarginatus	9	5.3	0.048		0.034
	Aphaenogaster fulva	Tenomerga cinerea	4	1.7	0.016		0.034
	Hypoponera opacior	Sunius confluentus	6	2.9	0.026		0.036
	Nylanderia faisonensis	Apenes sinuatus	3	1	0.009		0.036
	Nylanderia faisonensis	Staphylinidae sp. 191	3	1	0.009		0.036
	Myrmecina americana	Thoracophorus costalis	6	3.2	0.03		0.037
	Pheidole dentata	Paratachys	5	2.2	0.02		0.038
	Myrmecina americana	Toxidium compressum	4	1.5	0.014		0.039
	Pheidole dentata	Lobrathium	4	1.5	0.014		0.039
	Monomorium minimum	Buprestis lineata	13	9.3	0.084		0.042
	Camponotus chromaoides	Vanonus	5	2.2	0.02		0.042
	Lasius alienus	Aglyptinus laevis	5	2.2	0.02		0.042
	Camponotus subbarbatus	Conalia helva	3	1.1	0.01		0.042
	Nylanderia faisonensis	Elateridae sp. 95	4	1.7	0.015		0.043
	Nylanderia faisonensis	Staphylinidae sp. 219	4	1.7	0.015		0.043
	Lasius alienus	Pycnomerus haematodes	10	6.4	0.058		0.045
	Camponotus subbarbatus	Plateros volatus	7	3.9	0.035		0.045
	Camponotus subbarbatus	Pycnoplectus	7	3.9	0.035		0.045
	Aphaenogaster lamellidans	Glipostenoda ambusta	4	1.5	0.014		0.046
	Pheidole dentata	Hymenorus	8	4.6	0.042		0.048
	Nylanderia faisonensis	Mioptachys flavicauda	31	26.9	0.245		0.049
	Aphaenogaster fulva	Clivina pallida	10	6.5	0.06		0.049
beetles x beetles	Priocera castanea	Uloma punctulata	1	7.7	0.07	0.000	
	Rhyncolus	Ampedus luteolus	4	14	0.127	0.000	
	Rhyncolus	Thoracophorus costalis	16	26.4	0.24	0.000	
	Cossonus corticola	Eblisia carolina	10	16.4	0.149	0.001	
	Rhyncolus	Leptusa	31	38.9	0.353	0.001	
	Vanonus	Pycnomerus haematodes	1	6.4	0.058	0.002	
	Priocera castanea	Sepedophilus debilis	3	8.8	0.08	0.002	
	Rhyncolus	Staphylinidae sp. 199	0	4.1	0.038	0.002	
	Rhyncolus	Baeocera pallida	4	10.4	0.094	0.002	

	Ganascus ventricosus	Uloma punctulata	1	6	0.054	0.003	
	Clivina pallida	Pycnomerus haematodes	1	6	0.055	0.003	
	Dryopthorus americanus	Bacanius sp. 2	0	3	0.027	0.003	
	Thoracophorus costalis	Xylopinus saperdiodes	3	8.8	0.08	0.003	
	Euvrilleta	Leptusa	2	6.1	0.056	0.004	
	Ganascus ventricosus	Sepedophilus debilis	2	6.9	0.062	0.005	
	Diplocoelus rudis	Rhyncolus	0	3.6	0.033	0.005	
	Buprestis lineata	Ampedus luteolus	0	4.2	0.038	0.005	
	Priocera castanea	Leptusa	7	12.3	0.112	0.005	
	Priocera castanea	Thoracophorus costalis	3	8.3	0.076	0.005	
	Vanonus	Ciidae	1	5.7	0.052	0.006	
	Chalcophora virginiensis	Rhyncolus	1	5.2	0.047	0.006	
	Vanonus	Oxycopis thoracica	0	3.9	0.036	0.007	
	Ampedus luteolus	Pycnomerus haematodes	5	10.8	0.098	0.007	
	Micromalthus debilis	Sepedophilus debilis	4	9.3	0.085	0.007	
	Clivina pallida	Rhyncolus	3	7.8	0.071	0.008	
	Dryopthorus americanus	Hymenorus	11	15.8	0.144	0.009	
	Rhyncolus	Rushia longula	0	3.1	0.028	0.011	
	Leptusa	Pycnomerus haematodes	24	30	0.273	0.011	
	Cossonus corticola	Staphylinidae sp. 199	3	6.3	0.057	0.012	
	Buprestis lineata	Bacanius sp. 1	0	3.6	0.032	0.013	
	Priocera castanea	Microscydmus sp. 2	1	5.1	0.046	0.014	
	Staphylinidae sp. 199	Pycnomerus haematodes	0	3.2	0.029	0.014	
	Glipostenoda ambusta	Thoracophorus costalis	2	6	0.055	0.016	
	Aleocharinae sp.	Pycnomerus relfexus	1	4.9	0.045	0.016	
	Euvrilleta	Sepedophilus debilis	1	4.4	0.04	0.018	
	Ptiliidae sp.	Sepedophilus debilis	1	4.4	0.04	0.018	
	Buprestis lineata	Microscydmus sp. 2	1	4.8	0.044	0.019	
	Rhyncolus	Bacanius sp. 1	7	11.9	0.108	0.019	
	Buprestis lineata	Eblisia carolina	0	3.2	0.03	0.020	
	Buprestis lineata	Uloma punctulata	3	7.3	0.066	0.020	
	Cossonus corticola	Melba thoracica	8	11.7	0.107	0.020	
	Drapetes quadripustulatus	Ampedus luteolus	0	3.2	0.029	0.020	
	Ampedus luteolus	Glipostenoda ambusta	0	3.2	0.029	0.020	
	Ciidae	Thesiastes	1	4.6	0.042	0.021	

	Lacon discoidea	Microscydmus sp. 2	0	3.1	0.028	0.021	
	Dryopthorus americanus	Rhyncolus	38	43	0.391	0.022	
	Rhyncolus	Pityobius anguinus	0	2.6	0.024	0.023	
	Rhyncolus	Xanthochroa lateralis	0	2.6	0.024	0.023	
	Holostrophus bifasciatus	Pycnomerus relfexus	1	4.6	0.042	0.023	
	Cossonus corticola	Uloma punctulata	32	36.7	0.334	0.024	
	Hymenorus	Pycnomerus haematodes	4	8.4	0.076	0.024	
	Priocera castanea	Euplectus	1	4.6	0.042	0.026	
	Rhyncolus	Microscydmus sp. 2	11	16.1	0.146	0.026	
	Clivina punctigera	Leptusa	0	2	0.019	0.030	
	Cossonus corticola	Microscydmus sp. 2	20	24.2	0.22	0.030	
	Cossonus corticola	Uloma imberbis	9	12.5	0.114	0.030	
	Rhyncolus	Uloma punctulata	19	24.4	0.221	0.030	
	Priocera castanea	Ampedus luteolus	1	4.4	0.04	0.031	
	Dicerca tenebrosa knulli	Sepedophilus debilis	1	3.9	0.036	0.034	
	Vanonus	Xylopinus saperdiodes	0	2.8	0.025	0.037	
	Priocera castanea	Tmesiphorus carinatus	0	2.8	0.025	0.037	
	Cossonus corticola	Toxidium compressum	15	18.8	0.171	0.038	
	Lacon discoidea	Ampedus luteolus	0	2.7	0.025	0.038	
	Lacon discoidea	Oxycopis thoracica	0	2.7	0.025	0.038	
	Chalcophora virginiensis	Pycnomerus haematodes	1	4	0.036	0.039	
	Ganascus ventricosus	Thoracophorus costalis	3	6.5	0.059	0.041	
	Philothermus glabriculus	Eustrophopsis bicolor	1	3.7	0.034	0.041	
	Eblisia carolina	Sunius confluentus	2	5.5	0.05	0.041	
	Ganascus ventricosus	Eblisia carolina	0	2.7	0.024	0.042	
	Staphylinidae sp. 211	Pycnomerus haematodes	0	2.4	0.022	0.042	
	Euvrilleta	Uloma punctulata	1	3.8	0.035	0.044	
	Dryopthorus americanus	Lycidae sp. 104	1	3	0.027	0.045	
	Cossonus corticola	Platydema ruficorne	0	1.6	0.014	0.046	
	Clivina pallida	Xylopinus saperdiodes	0	2.6	0.024	0.047	
	Ciidae	Lacon discoidea	1	3.9	0.035	0.048	
	Rhyncolus	Eblisia carolina	7	10.9	0.099	0.050	
	Vanonus	Lacon impressicollis	6	1.3	0.012		0.000
	Ganascus ventricosus	Xylopinus saperdiodes	8	2.4	0.022		0.000
	Chalcophora virginiensis	Clivina pallida	6	1.4	0.012		0.000

	Chalcophora virginiensis	Ampedus luteolus	9	2.5	0.022		0.000
	Paratachys	Polyderis laevis	9	1.8	0.017		0.000
	Polyderis laevis	Palaminus	6	1.1	0.01		0.000
	Polyderis laevis	Lobrathium	6	1.3	0.012		0.000
	Polyderis laevis	Microscydmus sp. 1	7	1.3	0.012		0.000
	Philothermus glabriculus	Thoracophorus costalis	36	26.9	0.244		0.000
	Priocera castanea	Rhyncolus	16	9.3	0.085		0.000
	Rhyncolus	Drapetes quadripustulatus	13	6.7	0.061		0.000
	Rhyncolus	Glipostenoda ambusta	13	6.7	0.061		0.000
	Rhyncolus	Pycnomerus haematodes	34	22.8	0.207		0.000
	Drapetes quadripustulatus	Glipostenoda ambusta	8	1.5	0.014		0.000
	Eblisia carolina	Euplectus	13	5.3	0.049		0.000
	Eblisia carolina	Uloma punctulata	18	9	0.082		0.000
	Glipostenoda ambusta	Oxycopis thoracica	9	3.2	0.029		0.000
	Leptusa	Thoracophorus costalis	44	34.8	0.316		0.000
	Microscydmus sp. 2	Uloma punctulata	23	13.2	0.12		0.000
	Euplectus	Uloma punctulata	21	12	0.109		0.000
	Philothermus glabriculus	Leptusa	48	39.5	0.36		0.001
	Drapetes quadripustulatus	Xylopinus saperdiodes	7	2.2	0.02		0.001
	Ampedus luteolus	Bacanius sp. 1	12	5.6	0.051		0.001
	Ampedus luteolus	Xanthochroa lateralis	5	1.2	0.011		0.001
	Ampedus luteolus	Staphylinidae sp. 168	5	1.2	0.011		0.001
	Ampedus luteolus	Leptusa	25	18.4	0.167		0.001
	Ampedus luteolus	Thoracophorus costalis	20	12.5	0.114		0.001
	Ampedus luteolus	Uloma punctulata	19	11.5	0.105		0.001
	Eblisia carolina	Staphylinidae sp. 202	6	1.7	0.016		0.001
	Glipostenoda ambusta	Xylopinus saperdiodes	7	2.2	0.02		0.001
	Glipostenoda ambusta	Pycnomerus haematodes	11	5.2	0.047		0.001
	Sepedophilus debilis	Leptusa	45	36.8	0.335		0.001
	Leptusa	Uloma punctulata	40	32	0.291		0.001
	Staphylinidae sp. 189	Anacyptus testaceus	6	1.5	0.014		0.001
	Microscydmus sp. 2	Staphylinidae sp. 219	5	1.4	0.013		0.001
	Xylopinus saperdiodes	Pycnomerus haematodes	14	7.6	0.069		0.001
	Clivina pallida	Ampedus luteolus	9	3.7	0.033		0.002
	Polyderis laevis	Sunius confluentus	11	5.3	0.048		0.002

	Philothermus glabriculus	Ampedus luteolus	21	14.2	0.129		0.002
	Bacanius sp. 1	Pycnomerus relfexus	14	7.5	0.068		0.002
	Microscydmus sp. 2	Toxidium compressum	13	6.8	0.061		0.002
	Dryopthorus americanus	Leptusa	63	56.6	0.514		0.003
	Oxycopis thoracica	Xylopinus saperdiodes	10	4.7	0.042		0.003
	Sepedophilus debilis	Sunius confluentus	21	14.2	0.129		0.003
	Drapetes quadripustulatus	Plateros volatus	5	1.3	0.012		0.003
	Drapetes quadripustulatus	Oxycopis thoracica	8	3.2	0.029		0.003
	Oxycopis thoracica	Platydema nigratum	5	1.5	0.013		0.003
	Bacanius sp. 1	Uloma punctulata	16	9.8	0.089		0.004
	Sunius confluentus	Thoracophorus costalis	20	13.4	0.122		0.004
	Leptusa	Euplectus	25	19.1	0.174		0.004
	Melba thoracica	Toxidium compressum	8	3.3	0.03		0.004
	Rhyncolus	Staphylinidae sp. 193	8	4.1	0.038		0.004
	Lycidae sp. 104	Sunius confluentus	4	1.1	0.01		0.004
	Plateros volatus	Pycnomerus haematodes	9	4.4	0.04		0.004
	Buprestis lineata	Glipostenoda ambusta	6	2	0.018		0.005
	Drapetes quadripustulatus	Pycnomerus haematodes	10	5.2	0.047		0.005
	Cossonus corticola	Oxycopis thoracica	26	21.1	0.192		0.005
	Ciidae	Eblisia carolina	13	7.4	0.068		0.006
	Eblisia carolina	Staphylinidae sp. 199	5	1.5	0.014		0.006
	Sepedophilus debilis	Euplectus	20	13.7	0.125		0.006
	Leptusa	Microscydmus sp. 2	27	21.1	0.192		0.006
	Rhyncolus	Plateros volatus	10	5.7	0.052		0.006
	Buprestis lineata	Priocera castanea	7	2.8	0.025		0.007
	Typocerus zebra	Micromalthus debilis	5	1.6	0.014		0.007
	Priocera castanea	Pycnomerus relfexus	11	5.9	0.054		0.007
	Ampedus luteolus	Staphylinidae sp. 202	6	2.2	0.02		0.007
	Micromalthus debilis	Staphylinidae sp. 202	5	1.6	0.014		0.007
	Eblisia carolina	Microscydmus sp. 2	11	5.9	0.054		0.008
	Uloma imberbis	Pycnomerus relfexus	10	5.2	0.048		0.008
	Pseudariotus notatus	Xylopinus saperdiodes	4	1	0.009		0.008
	Eblisia carolina	Melba thoracica	7	2.9	0.026		0.008
	Priocera castanea	Cossonus corticola	18	14.1	0.128		0.008
	Palaminus	Thoracophorus costalis	6	2.8	0.025		0.008

	Philothermus glabriculus	Oxycopis thoracica	20	14.2	0.129		0.009
	Ciidae	Toxidium compressum	14	8.5	0.077		0.009
	Ampedus luteolus	Eblisia carolina	10	5.2	0.047		0.009
	Plateros volatus	Staphylinidae sp. 189	4	1	0.009		0.009
	Ampedus luteolus	Aglyptinus laevis	8	3.7	0.033		0.010
	Carabidae sp. 90	Polyderis laevis	4	1.1	0.01		0.010
	Ciidae	Plateros volatus	8	3.9	0.035		0.010
	Lacon impressicollis	Sunius confluentus	6	2.4	0.022		0.010
	Staphylinidae sp. 199	Uloma punctulata	7	3.4	0.031		0.010
	Buprestis lineata	Cossonus corticola	17	13.3	0.121		0.010
	Dryopthorus americanus	Aglyptinus laevis	15	11.3	0.103		0.010
	Mioptachys flavicauda	Philothermus glabriculus	48	42.2	0.383		0.011
	Dryopthorus americanus	Uloma punctulata	41	35.5	0.322		0.011
	Bacanius sp. 1	Thoracophorus costalis	16	10.7	0.097		0.011
	Ganascus ventricosus	Priocera castanea	6	2.3	0.021		0.011
	Eblisia carolina	Leptusa	19	14.3	0.13		0.011
	Aglyptinus laevis	Uloma punctulata	11	6.4	0.058		0.011
	Sepedophilus debilis	Microscydmus sp. 2	21	15.2	0.138		0.012
	Ciidae	Oxycopis thoracica	15	9.6	0.087		0.012
	Dryopthorus americanus	Ampedus luteolus	25	20.4	0.185		0.012
	Lacon impressicollis	Hymenorus	5	1.7	0.016		0.012
	Staphylinidae sp. 199	Melba thoracica	4	1.1	0.01		0.012
	Staphylinidae sp. 199	Toxidium compressum	5	1.7	0.016		0.012
	Pycnoplectus	Anacyptus testaceus	5	1.7	0.015		0.013
	Pityobius anguinus	Ampedus luteolus	4	1.2	0.011		0.013
	Microscydmus sp. 2	Euplectus	13	7.9	0.072		0.014
	Eblisia carolina	Toxidium compressum	9	4.6	0.042		0.014
	Oxycopis thoracica	Staphylinidae sp. 189	6	2.5	0.022		0.014
	Microscydmus sp. 2	Scaphisoma sp. 1	6	2.5	0.023		0.014
	Dryopthorus americanus	Holostrophus bifasciatus	14	10.6	0.096		0.014
	Bacanius sp. 1	Aglyptinus laevis	7	3.1	0.029		0.015
	Staphylinidae sp. 189	Uloma punctulata	8	4.3	0.039		0.015
	Staphylinidae sp. 199	Uloma imberbis	4	1.2	0.011		0.015
	Scaphisoma sp. 1	Holostrophus bifasciatus	4	1.1	0.01		0.015
	Clivina pallida	Sunius confluentus	8	4	0.036		0.016

	Leptusa	Toxidium compressum	21	16.4	0.149		0.016
	Aleocharinae sp.	Thesiastes	5	1.8	0.016		0.016
	Oxycopis thoracica	Pycnomerus haematodes	16	10.8	0.098		0.017
	Mioptachys flavicauda	Euplectus	25	20.4	0.185		0.017
	Polyderis laevis	Paromalus	5	1.8	0.017		0.017
	Xanthochroa lateralis	Sunius confluentus	4	1.3	0.012		0.017
	Sepedophilus debilis	Uloma punctulata	29	23.1	0.21		0.018
	Pycnomerus haematodes	Pycnomerus relfexus	20	14.4	0.131		0.018
	Polyderis laevis	Thoracophorus costalis	14	9.3	0.084		0.018
	Philothermus glabriculus	Bacanius sp. 1	17	12.1	0.11		0.019
	Tmesiphorus carinatus	Hymenorus	7	3.2	0.03		0.019
	Euvrilleta	Aleocharinae sp.	4	1.2	0.011		0.019
	Dicerca tenebrosa knulli	Buprestis lineata	4	1.2	0.011		0.019
	Clivina pallida	Leptusa	14	10.2	0.093		0.019
	Clivina pallida	Pycnomerus sulcicollis	4	1.2	0.011		0.019
	Piesmus submarginatus	Scaphisoma sp. 1	4	1.2	0.011		0.019
	Staphylinidae sp. 199	Tmesiphorus carinatus	4	1.2	0.011		0.019
	Xanthochroa lateralis	Thoracophorus costalis	5	2.3	0.021		0.019
	Eblisia carolina	Hymenorus	8	4	0.036		0.020
	Typocerus zebra	Pycnomerus haematodes	7	3.6	0.033		0.020
	Diplocoelus rudis	Baeocera pallida	4	1.3	0.012		0.020
	Staphylinidae sp. 211	Toxidium compressum	4	1.3	0.012		0.020
	Dryopthorus americanus	Thesiastes	13	9.8	0.089		0.020
	Thoracophorus costalis	Toxidium compressum	16	11.1	0.101		0.021
	Bacanius sp. 1	Oxycopis thoracica	10	5.6	0.051		0.021
	Ampedus luteolus	Staphylinidae sp. 199	5	2	0.018		0.021
	Plateros volatus	Xylopinus saperdiodes	5	1.9	0.017		0.021
	Euvrilleta	Rhyncolus	8	4.7	0.042		0.021
	Mioptachys flavicauda	Dryopthorus americanus	65	60.4	0.549		0.022
	Ciidae	Staphylinidae sp. 189	7	3.5	0.032		0.022
	Polyderis laevis	Ampedus luteolus	9	4.9	0.045		0.023
	Uloma imberbis	Uloma punctulata	11	6.8	0.062		0.023
	Piesmus submarginatus	Holostrophus bifasciatus	5	1.9	0.017		0.023
	Ciidae	Microscydmus sp. 2	16	11	0.1		0.024
	Euplectus	Thoracophorus costalis	18	13	0.118		0.024

	Cossonus corticola	Pycnomerus haematodes	39	34.4	0.313		0.024
	Buprestis lineata	Rhyncolus	13	8.8	0.08		0.024
	Buprestis lineata	Pycnomerus haematodes	11	6.8	0.062		0.024
	Anacyptus testaceus	Pycnomerus haematodes	11	6.8	0.062		0.024
	Clivina pallida	Thoracophorus costalis	11	7	0.063		0.024
	Tmesiphorus carinatus	Anacyptus testaceus	6	2.6	0.024		0.024
	Thoracophorus costalis	Pycnomerus relfexus	22	16.7	0.152		0.025
	Piesmus submarginatus	Microscydmus sp. 2	8	4.2	0.038		0.025
	Ampedus luteolus	Tmesiphorus carinatus	8	4.2	0.038		0.025
	Melanotus ignobilis	Ampedus luteolus	6	2.7	0.025		0.025
	Plateros volatus	Oxycopis thoracica	6	2.7	0.025		0.025
	Vanonus	Scaphisoma sp. 1	4	1.3	0.012		0.025
	Plateros volatus	Glipostenoda ambusta	4	1.3	0.012		0.025
	Mioptachys flavicauda	Melanotus ignobilis	11	8	0.073		0.025
	Mioptachys flavicauda	Microscydmus sp. 2	27	22.5	0.205		0.026
	Eblisia carolina	Holostrophus bifasciatus	6	2.7	0.024		0.026
	Leptusa	Staphylinidae sp. 202	9	6.1	0.056		0.027
	Bacanius sp. 1	Euplectus	10	5.9	0.053		0.028
	Philothermus glabriculus	Tmesiphorus carinatus	13	9	0.081		0.029
	Polyderis laevis	Aglyptinus laevis	6	2.7	0.025		0.029
	Buprestis lineata	Drapetes quadripustulatus	5	2	0.018		0.029
	Melanotus ignobilis	Euplectus	6	2.8	0.025		0.030
	Ampedus luteolus	Palaminus	4	1.5	0.013		0.031
	Clivina pallida	Aglyptinus laevis	5	2	0.019		0.032
	Chalcophora virginiensis	Bacanius sp. 1	5	2.1	0.019		0.032
	Bacanius sp. 1	Staphylinidae sp. 189	5	2.1	0.019		0.032
	Ampedus luteolus	Hymenorus	9	5.2	0.047		0.033
	Typocerus zebra	Pycnomerus relfexus	6	2.9	0.027		0.033
	Cossonus corticola	Glipostenoda ambusta	13	10.2	0.092		0.033
	Thoracophorus costalis	Uloma punctulata	27	21.8	0.198		0.034
	Polyderis laevis	Sepedophilus debilis	14	9.8	0.089		0.034
	Melanotus ignobilis	Eblisia carolina	5	2.1	0.019		0.034
	Pycnoplectus	Hymenorus	5	2.1	0.019		0.034
	Mioptachys flavicauda	Staphylinidae sp. 189	10	7.3	0.066		0.035
	Mioptachys flavicauda	Leptusa	59	54.5	0.496		0.036

	Dryopthorus americanus	Thoracophorus costalis	43	38.5	0.35		0.036
	Melanotus ignobilis	Uloma punctulata	8	4.7	0.043		0.036
	Priocera castanea	Drapetes quadripustulatus	5	2.1	0.019		0.037
	Priocera castanea	Glipostenoda ambusta	5	2.1	0.019		0.037
	Dryopthorus americanus	Bacanius sp. 1	21	17.4	0.158		0.037
	Philothermus glabriculus	Xanthochroa lateralis	5	2.6	0.024		0.037
	Dryopthorus americanus	Euplectus	25	21.1	0.192		0.038
	Piesmus submarginatus	Eblisia carolina	6	2.9	0.026		0.038
	Piesmus submarginatus	Hymenorus	6	2.9	0.026		0.038
	Paromalus	Toxidium compressum	5	2.2	0.02		0.038
	Euvrilleta	Priocera castanea	4	1.5	0.013		0.038
	Typocerus zebra	Priocera castanea	4	1.5	0.013		0.038
	Dicerca tenebrosa knulli	Rhyncolus	7	4.1	0.038		0.038
	Euplectus	Toxidium compressum	10	6.1	0.056		0.039
	Sunius confluentus	Baeocera pallida	9	5.3	0.048		0.039
	Aglyptinus laevis	Oxycopis thoracica	7	3.7	0.033		0.039
	Ampedus luteolus	Pycnomerus sulcicollis	5	2.2	0.02		0.039
	Staphylinidae sp. 168	Pycnomerus relfexus	4	1.6	0.015		0.039
	Leptusa	Pycnomerus relfexus	29	24.5	0.223		0.040
	Aglyptinus laevis	Sepedophilus debilis	11	7.4	0.067		0.040
	Microscydmus sp. 2	Uloma imberbis	8	4.5	0.041		0.040
	Ciidae	Glipostenoda ambusta	8	4.6	0.042		0.040
	Thesiastes	Uloma punctulata	9	5.6	0.05		0.040
	Ciidae	Baeocera pallida	11	7.1	0.064		0.041
	Melanotus sp. 3	Sunius confluentus	4	1.6	0.014		0.041
	Melba thoracica	Uloma punctulata	10	6.4	0.058		0.042
	Piesmus submarginatus	Melanotus ignobilis	4	1.5	0.014		0.042
	Ciidae	Cathartosilvanus imbellis	3	1.1	0.01		0.042
	Ciidae	Staphylinidae sp. 180	3	1.1	0.01		0.042
	Tmesiphorus carinatus	Uloma punctulata	11	7.3	0.066		0.043
	Micromalthus debilis	Anacyptus testaceus	6	2.9	0.027		0.043
	Leptusa	Hymenorus	18	14.3	0.13		0.044
	Dicerca tenebrosa knulli	Pycnomerus haematodes	6	3.2	0.029		0.044
	Staphylinidae sp. 193	Pycnomerus haematodes	6	3.2	0.029		0.044
	Mioptachys flavicauda	Aleocharinae sp.	14	10.9	0.099		0.044

	Cossonus corticola	Micromalthus debilis	18	14.9	0.135		0.044
	Cossonus corticola	Xylopinus saperdiodes	18	14.9	0.135		0.044
	Ciidae	Pycnoplectus	7	3.9	0.035		0.045
	Euplectus	Scaphisoma sp. 1	5	2.3	0.021		0.045
	Euplectus	Pycnomerus sulcicollis	5	2.3	0.021		0.045
	Buprestis lineata	Paromalus	4	1.5	0.014		0.046
	Paratachys	Tmesiphorus carinatus	4	1.5	0.014		0.046
	Typocerus zebra	Xylopinus saperdiodes	4	1.6	0.014		0.046
	Aglyptinus laevis	Euplectus	7	3.8	0.035		0.048
	Philothermus glabriculus	Dryopthorus americanus	48	43.8	0.398		0.049
	Ciidae	Staphylinidae sp. 202	6	3.2	0.029		0.049
	Philothermus glabriculus	Euplectus	19	14.8	0.134		0.050

Supplemental Table S2. Co-occurrence results for the 180 species included in co-occurrence analysis showing what percentage of total pairings were positive negative or neutral.

Order	Taxon	Family	total pairings	% positive	% negative	% neutral
Blattodea (termites)	Reticulitermes	Rhinotermitidae	179	0.56	3.35	96.09
Hymenoptera (ants)	Aphaenogaster carolinensis	Formicidae	97	0	4.12	95.88
	Aphaenogaster fulva	Formicidae	142	2.82	2.11	95.07
	Aphaenogaster lamellidans	Formicidae	75	2.67	0	97.33
	Camponotus castaneus	Formicidae	35	2.86	2.86	94.29
	Camponotus chromaoides	Formicidae	78	3.85	3.85	92.31
	Camponotus subbarbatus	Formicidae	142	5.63	4.23	90.14
	Crematogaster ashmeadi	Formicidae	23	0	0	100
	Crematogaster cerasi	Formicidae	23	0	0	100
	Crematogaster lineolata	Formicidae	9	0	0	100
	Cryptopone gilva	Formicidae	17	0	0	100
	Dorymyrmex flavus	Formicidae	9	0	11.11	88.89
	Hypoponera opaciceps	Formicidae	9	0	0	100
	Hypoponera opacior	Formicidae	65	3.08	0	96.92
	Lasius alienus	Formicidae	86	2.33	1.16	96.51
	Monomorium minimum	Formicidae	179	2.23	1.68	96.09
	Myrmecina americana	Formicidae	42	4.76	4.76	90.48
	Nylanderia faisonensis	Formicidae	142	12.68	1.41	85.92
	Nylanderia querna	Formicidae	65	3.08	0	96.92
	Pheidole dentata	Formicidae	106	5.66	1.89	92.45
	Pheidole navigans	Formicidae	23	0	4.35	95.65
	Ponera exotica	Formicidae	17	0	0	100
	Ponera pennsylvanica	Formicidae	78	8.97	0	91.03
	Proceratium croceum	Formicidae	42	7.14	0	92.86
	Proceratium silaceum	Formicidae	56	1.79	0	98.21
	Solenopsis geminata	Formicidae	42	0	4.76	95.24
	Solenopsis molesta	Formicidae	179	0	2.79	97.21
	Strumigenys clypeata	Formicidae	35	2.86	0	97.14
	Strumigenys louisianae	Formicidae	56	5.36	1.79	92.86
	Strumigenys ornata	Formicidae	17	0	0	100
	Strumigenys rostrata	Formicidae	121	5.79	1.65	92.56

	Temnothorax curvispinosus	Formicidae	121	0.83	0.83	98.35
	Temnothorax pergandei	Formicidae	9	0	0	100
	Temnothorax schaumii	Formicidae	9	0	0	100
Coleoptera (beetles)	Adranes	Staphylinidae	17	0	0	100
	Aeletes sp. 1	Histeridae	28	0	0	100
	Agathidium	Leiodidae	9	0	0	100
	Aglyptinus laevis	Leiodidae	78	14.1	0	85.9
	Aleocharinae sp.	Staphylinidae	78	3.85	1.28	94.87
	Ampedus luteolus	Elateridae	106	20.75	6.6	72.64
	Ampedus rubricollis	Elateridae	9	0	0	100
	Anacyptus testaceus	Staphylinidae	86	5.81	1.16	93.02
	Anisotoma sp. 1	Leiodidae	9	0	0	100
	Apenes sinuatus	Carabidae	17	5.88	0	94.12
	Aulonothroscus convergens	Throscidae	35	0	0	100
	Bacanius sp. 1	Histeridae	106	10.38	1.89	87.74
	Bacanius sp. 2	Histeridae	23	0	4.35	95.65
	Baeocera pallida	Staphylinidae	97	3.09	1.03	95.88
	Bitoma	Zopheridae	28	0	0	100
	Buprestis lineata	Buprestidae	86	11.63	5.81	82.56
	Caerosternus americanus	Histeridae	9	0	0	100
	Canifa pallipes	Scraptiidae	23	0	4.35	95.65
	Carabidae sp. 89	Carabidae	23	0	0	100
	Carabidae sp. 90	Carabidae	35	5.71	0	94.29
	Cathartosilvanus imbellis	Silvanidae	17	5.88	0	94.12
	Catogenus rufus	Passandridae	17	0	0	100
	Chalcophora virginiensis	Buprestidae	62	6.45	4.84	88.71
	Chrysomelidae sp. 110	Chrysomelidae	9	0	0	100
	Ciidae	Ciidae	142	8.45	2.82	88.73
	Clivina americana	Carabidae	17	0	0	100
	Clivina pallida	Carabidae	78	14.1	3.85	82.05
	Clivina punctigera	Carabidae	17	0	5.88	94.12
	Conalia helva	Mordellidae	17	5.88	5.88	88.24
	Coproporus ventriculus	Staphylinidae	9	0	0	100
	Corylophidae sp. 142	Corylophidae	17	0	0	100
	Cossonus corticola	Curculionidae	179	5.59	5.03	89.39

	Cymindus limbatus	Carabidae	9	0	0	100
	Dicerca tenebrosa knulli	Buprestidae	51	5.88	1.96	92.16
	Dioedus punctatus	Tenebrionidae	42	0	0	100
	Diplocoelus rudis	Biphyllidae	42	2.38	4.76	92.86
	Drapetes quadripustulatus	Elateridae	75	10.67	1.33	88
	Dryopthorus americanus	Curculionidae	179	6.7	2.79	90.5
	Eblisia carolina	Histeridae	97	17.53	5.15	77.32
	Elateridae sp. 95	Elateridae	28	7.14	0	92.86
	Elateridae sp. 97	Elateridae	9	0	0	100
	Eucnemidae sp. 94	Eucnemidae	17	0	0	100
	Eumicrota	Staphylinidae	56	0	0	100
	Euplectus	Staphylinidae	121	13.22	0.83	85.95
	Eustrophopsis bicolor	Tetratomidae	42	0	4.76	95.24
	Euvrilleta	Anobiidae	56	5.36	7.14	87.5
	Ganascus ventricosus	Aderidae	78	2.56	6.41	91.03
	Glipostenoda ambusta	Mordellidae	75	14.67	4	81.33
	Helops aereus	Tenebrionidae	17	0	0	100
	Histeridae sp. 148	Histeridae	9	0	0	100
	Holostrophus bifasciatus	Tetratomidae	78	5.13	1.28	93.59
	Hymenorus	Tenebrionidae	97	9.28	2.06	88.66
	Lacon discoidea	Elateridae	65	0	6.15	93.85
	Lacon impressicollis	Elateridae	56	5.36	0	94.64
	Latridiidae sp. 129	Latridiidae	9	0	0	100
	Leptusa	Staphylinidae	179	8.94	2.79	88.27
	Lobrathium	Staphylinidae	42	7.14	2.38	90.48
	Lycidae sp. 104	Lycidae	23	8.7	4.35	86.96
	Melanotus ignobilis	Elateridae	65	9.23	0	90.77
	Melanotus sp. 1	Elateridae	75	0	0	100
	Melanotus sp. 3	Elateridae	35	2.86	0	97.14
	Melba thoracica	Staphylinidae	78	5.13	2.56	92.31
	Micromalthus debilis	Micromalthidae	97	4.12	1.03	94.85
	Microscydmus sp. 1	Staphylinidae	42	4.76	2.38	92.86
	Microscydmus sp. 2	Staphylinidae	121	10.74	4.13	85.12
	Microtonus sericans	Melandryidae	28	0	0	100
	Mioptachys flavicauda	Carabidae	179	5.59	1.12	93.3

	Mordellidae sp. 121	Mordellidae	9	0	0	100
	Neandra brunnea	Cerambycidae	17	0	0	100
	Nitidulidae sp. 132	Nitidulidae	17	0	0	100
	Omoglymmius americanus	Rhysodidae	9	0	0	100
	Orthosoma brunneum	Cerambycidae	9	0	0	100
	Oxycopis thoracica	Oedemeridae	106	11.32	2.83	85.85
	Palaminus	Staphylinidae	35	11.43	0	88.57
	Paratachys	Carabidae	62	6.45	1.61	91.94
	Paromalus	Histeridae	62	4.84	0	95.16
	Philothermus glabriculus	Cerylonidae	179	6.7	0.56	92.74
	Piesmus submarginatus	Carabidae	78	8.97	0	91.03
	Pityobius anguinus	Elateridae	28	7.14	3.57	89.29
	Plateros volatus	Lycidae	65	13.85	0	86.15
	Platydema flavipes	Tenebrionidae	35	0	0	100
	Platydema nigratum	Tenebrionidae	35	2.86	0	97.14
	Platydema ruficorne	Tenebrionidae	9	0	11.11	88.89
	Polyderis laevis	Carabidae	97	13.4	0	86.6
	Priocera castanea	Cleridae	86	11.63	10.47	77.91
	Pseudaptinus lecontei	Carabidae	9	0	0	100
	Pseudariotus notatus	Aderidae	35	2.86	0	97.14
	Ptiliidae sp.	Ptiliidae	56	0	1.79	98.21
	Pycnomerus haematodes	Zopheridae	142	9.86	6.34	83.8
	Pycnomerus relfexus	Zopheridae	121	6.61	1.65	91.74
	Pycnomerus sulcicollis	Zopheridae	56	7.14	0	92.86
	Pycnoplectus	Staphylinidae	65	6.15	0	93.85
	Rhyncolus	Curculionidae	179	5.59	10.61	83.8
	Rushia longula	Melandryidae	35	0	2.86	97.14
	Scaphisoma sp. 1	Staphylinidae	56	10.71	0	89.29
	Scaphisoma sp. 2	Staphylinidae	9	0	0	100
	Scarabaeidae sp. 85	Scarabaeidae	9	0	0	100
	Scolytinae sp. 118	Curculionidae	9	0	0	100
	Sepedophilus debilis	Staphylinidae	142	4.93	4.23	90.85
	Sepedophilus macer	Staphylinidae	17	0	5.88	94.12
	Sepedophilus sp. 2	Staphylinidae	23	0	0	100
	Silvanus muticus	Silvanidae	9	0	0	100

	Sphindidae	Sphindidae	23	4.35	0	95.65
	Staphylinidae sp. 162	Staphylinidae	23	4.35	0	95.65
	Staphylinidae sp. 163	Staphylinidae	9	0	0	100
	Staphylinidae sp. 164	Staphylinidae	9	0	0	100
	Staphylinidae sp. 168	Staphylinidae	28	7.14	0	92.86
	Staphylinidae sp. 170	Staphylinidae	9	0	0	100
	Staphylinidae sp. 171	Staphylinidae	17	0	0	100
	Staphylinidae sp. 178	Staphylinidae	23	4.35	0	95.65
	Staphylinidae sp. 180	Staphylinidae	17	5.88	0	94.12
	Staphylinidae sp. 182	Staphylinidae	17	0	0	100
	Staphylinidae sp. 185	Staphylinidae	23	0	0	100
	Staphylinidae sp. 189	Staphylinidae	62	12.9	0	87.1
	Staphylinidae sp. 191	Staphylinidae	17	5.88	0	94.12
	Staphylinidae sp. 193	Staphylinidae	51	5.88	0	94.12
	Staphylinidae sp. 194	Staphylinidae	9	0	0	100
	Staphylinidae sp. 195	Staphylinidae	9	0	0	100
	Staphylinidae sp. 197	Staphylinidae	9	0	0	100
	Staphylinidae sp. 199	Staphylinidae	51	15.69	7.84	76.47
	Staphylinidae sp. 202	Staphylinidae	56	8.93	0	91.07
	Staphylinidae sp. 204	Staphylinidae	9	0	0	100
	Staphylinidae sp. 207	Staphylinidae	9	0	0	100
	Staphylinidae sp. 208	Staphylinidae	9	0	0	100
	Staphylinidae sp. 209	Staphylinidae	9	0	0	100
	Staphylinidae sp. 211	Staphylinidae	35	2.86	2.86	94.29
	Staphylinidae sp. 212	Staphylinidae	23	0	0	100
	Staphylinidae sp. 214	Staphylinidae	28	3.57	0	96.43
	Staphylinidae sp. 217	Staphylinidae	17	0	0	100
	Staphylinidae sp. 219	Staphylinidae	28	7.14	0	92.86
	Stenus	Staphylinidae	9	0	0	100
	Sunius confluentus	Staphylinidae	121	9.09	1.65	89.26
	Tenomerga cinerea	Cupedidae	23	4.35	0	95.65
	Tharsus seditiosus	Tenebrionidae	9	0	0	100
	Thesiastes	Staphylinidae	75	4	1.33	94.67
	Thoracophorus costalis	Staphylinidae	142	10.56	4.23	85.21
	Tmesiphorus carinatus	Staphylinidae	86	9.3	2.33	88.37

	Toxidium compressum	Staphylinidae	106	12.26	0.94	86.79
	Typocerus zebra	Cerambycidae	56	8.93	0	91.07
	Uloma imberbis	Tenebrionidae	86	5.81	2.33	91.86
	Uloma punctulata	Tenebrionidae	142	12.68	4.93	82.39
	Vanonus	Aderidae	86	4.65	5.81	89.53
	Xanthochroa lateralis	Oedemeridae	28	14.29	3.57	82.14
	Xenistusa	Staphylinidae	9	0	0	100
	Xyloborus bispinatus	Curculionidae	23	0	4.35	95.65
	Xylopinus saperdiodes	Tenebrionidae	97	10.31	4.12	85.57
